# Plasma Polymerized Allylamine—The Unique Cell-Attractive Nanolayer for Dental Implant Materials

**DOI:** 10.3390/polym11061004

**Published:** 2019-06-05

**Authors:** J. Barbara Nebe, Henrike Rebl, Michael Schlosser, Susanne Staehlke, Martina Gruening, Klaus-Dieter Weltmann, Uwe Walschus, Birgit Finke

**Affiliations:** 1 Department of Cell Biology, Rostock University Medical Center, Schillingallee 69, 18057 Rostock, Germany; henrike.rebl@med.uni-rostock.de (H.R.); susanne.staehlke@med.uni-rostock.de (S.S.); martina.gruening@med.uni-rostock.de (M.G.); 2 Department Life, Light & Matter, University of Rostock, Albert-Einstein-Str. 25, 18059 Rostock, Germany; 3 Department of Surgery, University Medical Center Greifswald, 17475 Greifswald, Germany; schlosse@uni-greifswald.de; 4 Department of Medical Biochemistry and Molecular Biology, University Medical Center Greifswald, 17475 Greifswald, Germany; uwe.walschus@uni-greifswald.de; 5 Leibniz Institute for Plasma Science and Technology (INP), Felix-Hausdorff-Str. 2, 17489 Greifswald, Germany; weltmann@inp-greifswald.de (K.-D.W.); finke-hgw@t-online.de (B.F.)

**Keywords:** plasma polymerized allylamine, zirconia, surface characteristics, XPS, water contact angle, zeta potential, cell adhesion, osteoblasts, actin cytoskeleton, cell signaling

## Abstract

Biomaterials should be bioactive in stimulating the surrounding tissue to accelerate the ingrowth of permanent implants. Chemical and topographical features of the biomaterial surface affect cell physiology at the interface. A frequently asked question is whether the chemistry or the topography dominates the cell-material interaction. Recently, we demonstrated that a plasma-chemical modification using allylamine as a precursor was able to boost not only cell attachment and cell migration, but also intracellular signaling in vital cells. This microwave plasma process generated a homogenous nanolayer with randomly distributed, positively charged amino groups. In contrast, the surface of the human osteoblast is negatively charged at −15 mV due to its hyaluronan coat. As a consequence, we assumed that positive charges at the material surface—provoking electrostatic interaction forces—are attractive for the first cell encounter. This plasma-chemical nanocoating can be used for several biomaterials in orthopedic and dental implantology like titanium, titanium alloys, calcium phosphate scaffolds, and polylactide fiber meshes produced by electrospinning. In this regard, we wanted to ascertain whether plasma polymerized allylamine (PPAAm) is also suitable for increasing the attractiveness of a ceramic surface for dental implants using Yttria-stabilized tetragonal zirconia.

## 1. Introduction

Novel biomaterials in orthopedic or dental surgery should actively promote the osseointegration of implants. Both surface roughness and chemical modification can influence the interaction of osteoblasts with biomaterial surfaces. In implantology, titanium (Ti) and its alloys are often used because of their excellent biocompatibility and their mechanical properties. However, they are relatively inert and therefore new functionalization strategies are needed.

Consequently, it is essential to understand the initial reactions of cells at the material interface, including the organization of focal adhesion contacts that link the extracellular space and intracellular signaling processes. Human osteoblasts possess a net negative charge (−15 mV) [1] due to their hyaluronan coat [2], with carboxyl groups in the glycosaminoglycan chain. As a result, we assumed that positive charges at the material surface are attractive for the first cell attachment. We, therefore, developed a positively charged plasma polymerized nanolayer of allylamine (PPAAm) [2,3], which was deposited using physical low-pressure gas-discharge plasma in a microwave reactor. These processes are highly applicable, extremely effective, and valuable for the improvement of implant performance. There are, however, high demands in terms of process guidance and film quality—still a technological challenge even today. The PPAAm coating should adhere well and, thus, be mechanically stable; cross-linked and also stable in different aqueous media, resistant to hydrolysis and delamination, and equipped with a sufficient density of positively charged amino groups. Furthermore, the sterilizability of the plasma polymerized implant surfaces and their long-term storage characteristics are very important features, which have to be carefully evaluated before being used in implantology. For later applications, it was of interest to assess the applicability of this PPAAm layer on different material surfaces.

In recent years, we and other working groups [4,5,6,7,8,9] have thoroughly characterized this PPAAm layer containing amino groups in regard to its physico-chemical characteristics as well as cell biological outcomes after the modification of a variety of material substrates (Table 1) [10,11,12,13,14,15,16,17,18]. In general, this plasma polymer nanolayer showed cell-attractive characteristics that could not be achieved by coatings with extracellular matrix proteins, e.g., collagen type I or arginine-glycin-aspartic acid (Arg-Gly-Asp, RGD) (Table 1).

PPAAm was deposited on smooth and rough surfaces like polished, machined, or corundum-blasted titanium (Ti) [15] or titanium alloy [13,19], electrospun polylactide fiber meshes [14], β-tricalcium phosphate scaffolds [12], as well as a µm-thick, porous calcium phosphate layer [11]. The final plasma process conditions were chosen so as to yield substrate surfaces completely covered by a thin (>20 nm) PPAAm plasma polymer film. In this way, the chemical surface properties of the original substrate surface were completely replaced by the properties of PPAAm, whereby the mechanical properties of the bulk material were retained.

### 1.1. PPAAm Layer Characteristics—An Overview

Under the plasma-chemical deposition conditions [3,13,18,20] employed here, PPAAm films are mechanically stable and highly adhesive (80 N/mm^2^) [21]. A qualitative scratch test, a quantitative standard adhesive bonding strength test according to DIN EN 582 [22], and a wear test using artificial bone (30 pcf Sawbones and Palacos bone cement (PMMA)) [22] were carried out. An explanation for the excellent adhesion and robustness of the plasma polymer films deposited on zirconium/titanium substrates was given by the group of Bilek [23]: Metallic carbide and oxycarbide bonds were formed during the initial film formation by a two-dimensional-like, layer by layer (Frank-van der Merve) growth mode.

PPAAm coatings have, in general—regardless of the substrate—a positive zeta potential from +7.7 mV (pH 6) [18] to +8.6 mV (pH 7.4) [10] to +14 mV (pH 6) [16], and +26.3 mV (pH 6), also after long-term storage in ambient air for up to 200 days [13]. The total surface energy of the PPAAm film is 52 ± 8 mN/m, the polar part 22 ± 8 mN/m (Table 1). The XPS surface elemental composition of PPAAm is 30 ± 2% for N/C and near the theoretical N/C value of allylamine of 33%. O/C as a contamination is <10%. The amino group density NH_2_/C varies between 2–4%.

These surface properties and also the cell-attractive properties seem to be independent of the film thickness produced by different treatment times under the same pulse/pause ratio of the plasma.

The wettability of PPAAm-covered surfaces seems to be optimal for cell responses, with water contact angles (WCA) between 45°−68° (Table 1) [24]. Our deposited, very well cross-linked PPAAm films are stable in aqueous media [18]. This is a precondition for the layer effectiveness and stability in cell culture, in animal evaluations, or for later applications in human tissue.

### 1.2. In Vitro Outcome

The plasma-chemical modification with PPAAm of all these different biomaterial surfaces enormously improved initial cell adhesion, spreading, organization of adhesion-related components, and the cell ingrowth into the structured material surfaces (Table 1) [10,11,12,13,14,15,16,17,18]. The cells were strongly attached to the surface; this was detected by a spinning disk device [25]. Whereas the bone cell (MG-63) adherence was lowest on uncoated Ti6Al4V disks (44.2 ± 9.0 N/m^2^), on PPAAm-coated (66.8 ± 12.0 N/m^2^) and plasma polymerized ethylenediamine (PPEDA)-coated (53.2 ± 8.3 N/m^2^) Ti6Al4V disks, a significantly higher shear stress was necessary to detach the cells from the PPAAm/PPEDA surface, indicating a stronger cell attachment (after 18 h of cell growth).

**Figure 1:**
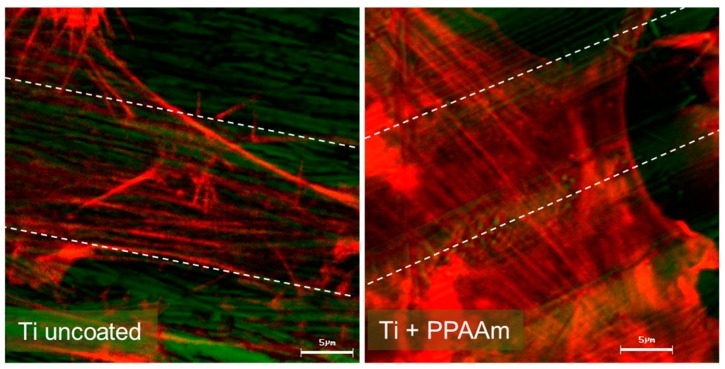
Cell alignment on stochastically structured Ti surfaces (Ti cp, grade 2, Ra 0.315 µm, [15]). Left: The actin filaments are aligned in the direction of the striations (dashed lines in grey) caused by machining. Right: Abrogated alignment due to PPAAm nanolayer coating of Ti (960 s gross) [15]. Technical description: Confocal laser scanning microscopy (LSM 410, Carl Zeiss AG, Oberkochen, Germany), scale bar 5 µm, actin staining (red) with Alexa Fluor 546 phalloidin, Ti surface in reflection mode false colored in green.

The osteoblasts displayed an extremely flattened phenotype and the cells seemed to merge with the topography of the surface. These highly cell-attractive characteristics of the PPAAm layer remain even after long-term storage up to one year or after γ-sterilization [13]. If cells grow on micro-grooved surfaces, the cells were aligned parallel to the grooves; this is called “contact guidance”. The functionalization of geometrically micro-grooved surfaces (20 µm groove width) with PPAAm resulted in an astonishing cell behavior: Although PPAAm is only a nanolayer, the cells were no longer able to “feel” the underlying grooves and the contact guidance was abrogated due to the chemistry on the surface [10]. We could observe this behavior also on PPAAm-coated, machined Ti surfaces: MG-63 cells could overcome the restrictions of the stochastically grooved surface and spread out over a large area [15]. However, the actin fibers of the cells on machined Ti + PPAAm were not arranged in parallel; instead, their actin fibers could also overcome the alignment (Figure 1).

**Figure 2:**
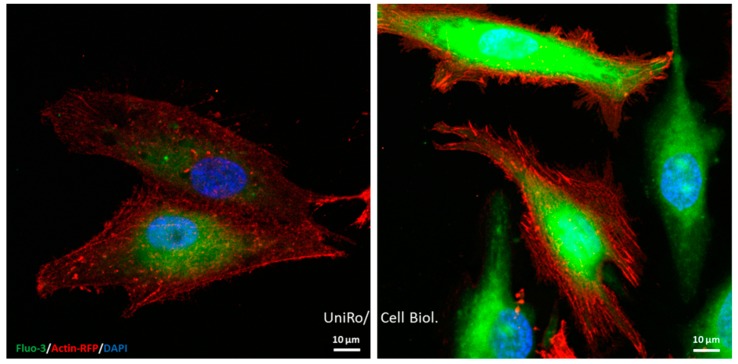
Basal calcium ion levels (green) in living MG-63 osteoblasts (red for actin, blue for nucleus) 24 h on planar Ti, either uncoated (**left**) or coated with a nanolayer of the plasma polymer (PPAAm, 480 s gross) (**right**). Note that on PPAAm, the intracellular basal Ca^2+^ ion level is elevated, indicating higher cellular activity. Live-cell staining: 50,000 cells/cm^2^ Ti wafer in DMEM with 10% FCS, staining with 20 µL BacMam 2.0 reagent (red) (actin-RFP, Life Technologies Corporation, Eugene, OR, USA) at 37 °C, 5 µM calcium indicator Fluo-3/AM (green) (Life Technologies Corporation) [27,28], and 1:1000 Hoechst 33342 (blue) (Thermo Fisher Scientific, Waltham, MA, USA) in isotonic HEPES buffer. Confocal laser scanning microscopy (LSM 780, Carl Zeiss Microscopy GmbH, Jena, Germany) with a plan-apochromat 63× oil immersion objective (Carl Zeiss, 1.40. Oil DIC M27), excitation at 405, 488, and 561 nm; scale bars 10 µm.

Our latest investigations by Moerke et al. [26] revealed that the restricted functions of osteoblasts on Ti micropillars (dimension 5 × 5 × 5 µm) are alleviated by nanocoating the surface with PPAAm. Human MG-63 cells growing on Ti micropillars normally express less mRNA of collagen type I, osteocalcin, and fibronectin after 24 h. This could be attenuated by coating of the micropillars with PPAAm. Although PPAAm is only a nanolayer, the influence was impressive and significant. At the same time, we saw cells very sensitive to the kinetics of calcium signaling on PPAAm-coated micropillars: After ATP stimulation, the intracellular calcium ion level increased impressively. The basal calcium ion content of cells on PPAAm was also significantly higher compared with uncoated Ti surfaces [26] as seen in the depiction (Figure 2). This is an indication that cells on PPAAm are very active in their cell physiology and it could explain why all the adhesion-related cell functions are accelerated.

### 1.3. In Vivo Outcome

The inflammatory response after implantation plays a central role for the in vivo biocompatibility of an implant, with macrophages and other immune cells being of central importance [29].

**Figure 3:**
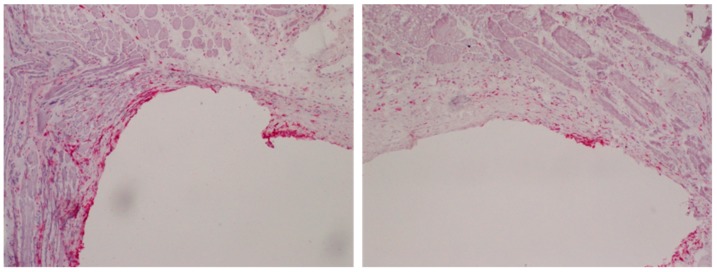
Total monocytes/macrophages (CD68^+^) in the peri-implant tissue 56 days after intramuscular implantation into rats of an unmodified Ti plate (**left**) vs. a PPAAm-coated Ti plate (**right**). Note the reduced macrophage response for PPAAm samples. Method: Peri-implant tissue samples were immediately shock-frozen after explantation and, following careful removal of the Ti plates, processed as cryosections (5 µm) and stained with monoclonal antibody ED1 (mouse anti-CD68); image magnification 125×.

To examine the influence of PPAAm films on the inflammatory response, Ti plates were coated with PPAAm and implanted intramuscularly in a rat model [30]. In this study, each animal simultaneously received an uncoated Ti implant and three Ti implants with PPAAm films created with different plasma process parameters. After 7, 14, and 56 days, the implants were removed together with the peri-implant tissue to quantify the number of total and tissue macrophages as well as total T-lymphocytes and MHC class II positive antigen-presenting cells following immunohistochemical staining. Based on experience from other studies, the different implantation periods were chosen to represent the acute phase of inflammation (day 7), the transition from acute to chronic inflammation (day 14), and the chronic phase of inflammation (day 56) typical for implant-related inflammation. On day 14, all three PPAAm-coated sample series demonstrated a significantly lower response of total macrophages than the uncoated samples. On day 56, the two-sample series that received a PPAAm variant with a shorter gross processing time still had a significantly lower number of total macrophages than the untreated samples (Figure 3). Taken together with the data from the physico-chemical analysis, these results indicate that different plasma process parameters lead to variations in the PPAAm film properties which influence the local inflammatory reactions.

Antibacterial surface modifications could prevent infections frequently associated with the implantation of biomaterials. In this regard, a surface modification with copper (Cu) could be an interesting approach. A surface treatment called plasma immersion ion implantation of copper (Cu-PIII) resulted in incorporation of copper ions into the topmost TiO_2_ layer. While implants treated with Cu-PIII had antibacterial properties in vitro, preliminary in vivo data revealed a stronger local inflammatory response after 56 days and a sustained increase of the IL-2 serum concentration. Thus, the hypothesis that an additional PPAAm film on Cu-PIII-treated Ti6Al4V samples could reduce these Cu-induced inflammatory reactions was tested in another study in rats. The results demonstrated that Cu-PIII-treated Ti6Al4V plates with an additional PPAAm coating induced local inflammatory tissue reactions comparable with untreated samples, while Cu-PIII-treated samples without PPAAm caused significantly stronger reactions in the chronic phase for several inflammatory cells [31].

Furthermore, the serum profile of different pro- and anti-inflammatory cytokines after implantation of Ti plates with PPAAm films or plasma polymerized acrylic acid films (PPAAc) in rats was examined to characterize the systemic inflammatory response. It was found that the different plasma modifications caused distinct serum cytokine profiles, which were as a whole comparable between the PPAAm samples and the untreated controls, while the PPAAc samples differed markedly from both PPAAm and controls [32]. The serum cytokine profile for the PPAAm samples also indicated two distinct phases of the systemic inflammatory response, possibly caused by time-dependent changes in the physico-chemical properties of PPAAm as described earlier [30]. The associations between the cytokine serum concentrations and different inflammatory cell populations were additionally examined by a multivariate correlation approach, revealing that high IFNγ concentrations in the early phase were associated with a high number of pro-inflammatory CD68^+^ monocyte/macrophages in the late phase [33]. This observation indicates that IFNγ could possibly be a serum marker to predict the local tissue response after implantation and associated implant-related complications.

In another in vivo approach using the rat femur, the osseointegration of Ti alloyed implants modified by PPAAm as well as plasma polymerized ethylenediamine (PPEDA) were evaluated [25]. Whereas uncoated Ti alloys showed the lowest bone-to-implant contact zone (BIC) (40.4%), the PPAAm and the PPEDA coatings revealed a clear increase of the BIC with 58.5% and 63.7%, respectively, indicating enhanced bone on growth [25].

We suggest that this amino-group-containing plasma polymer nanolayer (PPAAm) improves the cell–material interaction at the interface of diverse biomaterial surfaces, e.g., metals, polymers, calcium phosphates. Thus, we expect an improvement of the clinical outcome of permanent implants.

In this regard, we wanted to ascertain if PPAAm is also suitable to increase the attractiveness of a ceramic surface for dental implants. In recent years, these ceramic substrates were moved further into the center of healthcare, replacing metallic implant materials especially for patients with allergies. Building on our findings on PPAAm surface coatings of diverse bulk materials, the aim of our paper was to describe the interaction of MG-63 osteoblasts with very thin PPAAm films deposited on zirconia ceramic surfaces.

## 2. Materials and Methods

### 2.1. Substrates

Titanium: Disks of polished titanium (Ti), technical purity, cp grade 2 (Ra 0.2 µm) or titanium alloy (Ti6Al4V; Ra 0.4 µm), 11 mm in diameter and a height of 1 mm were used for comparison purposes as reference materials and for the chemical surface characteristics.

Furthermore, planar silicon (Si)-wafers (1 × 1 cm) were sputter-coated with 100 nm titanium (Ti, ZfM, Chemnitz, Germany) [10]. The resulting average surface roughness (Ra) was 0.774 nm, measured by atomic force microscopy (JPK Instruments, Berlin, Germany, [10]). The planar Si/Ti wafers were used to evaluate the optimal PPAAm plasma process duration and its resulting layer thickness on cell attachment.

Ceramics: Yttria-stabilized tetragonal zirconia polycrystals (Y-TZP, see patent) as a source material for dental implants were produced according to the same standard ISO 13356:1997. The material parameters such as density, the ratios of ZrO_2_, Y_2_O_3_, HfO_2_, and Al_2_O_3_, grain size, and the minimum bending strength are defined precisely by that norm. In this way, identical Y-TZP ceramic materials are ensured. Disks of Y-TZP, 13–20 mm in diameter and 2 mm thick with smooth or rough topographies (root-mean-squared-roughness Rq 273 ± 40 nm and Rq 1320 ± 20 nm, respectively) were utilized for the chemical functionalization, physico-chemical analysis, and in vitro cell culture. The roughnesses were determined with the profiler Dektak 3ST (Veeco Instruments, Santa Barbara, CA, USA; radius of the standard diamond stylus 2.5 mm, stylus force 30 mg, scan length 500 mm; 100 measurements/sample) (see Patent, chapter 5).

### 2.2. Deposition of Plasma Polymerized Allylamine (PPAAm)

Titanium: To determine the minimal possible layer thickness that allows cells to spread optimally, we evaluated the preconditions for the plasma deposition on the planar Ti wafers. The Ti wafers were coated by plasma polymerized allylamine (PPAAm) for 60 s (layer thickness <10 nm) or 480 s gross (layer thickness ~25 nm). Further preparation details are found in the following section. Ceramics: The microwave (MW) plasma reactor V55G (2.45 GHz; 60 L, Plasma Finish, Wertheim, Germany) was used for the deposition of the PPAAm films of different coating thicknesses. The substrates were in a downstream position 9 cm below the MW coupling window. First, the substrates were decontaminated and activated by continuous wave (cw) oxygen plasma (500 W, 50 Pa, 100 sccm O_2_, 300 s cw) and subsequently followed, without breaking the vacuum, by coating with PPAAm using allylamine as a precursor. A MW gas-discharge plasma (500 W) with a pulsed deposition regime (0.3 s on/1.7 s off) at low pressure (*p* = 50 Pa) was used for the coating. All samples were prepared under these identical standard plasma process conditions. Only the gross treatment time was varied from 960, 480, 240, 180, 120, 90, 60, 30, to 15 s [34]. Thin and very thin PPAAm films were realized dependent on the treatment time. A calibrated needle valve (0.125 ± 0.009 mL/min) allowed exact dosing of the monomer allylamine (H_2_C=CH-CH_2_-NH_2_) (VWR International GmbH, Darmstadt, Germany). Argon was used as a carrier gas (50 sccm).

### 2.3. Surface Characterization

#### 2.3.1. Grazing Incidence X-ray Diffraction (GIXRD), X-ray Photoelectron Spectroscopy (XPS), Fourier-Transform Infrared Spectroscopy FT-IR

GIXRD using a Siemens D5000 AXS diffractometer with Cu Kα radiation at 40 kV and 40 mA was carried out in order to study the crystallographic structure of the untreated and plasma-treated ceramic samples used. The measurements were performed at a constant incident angle of ω = 0.5° relative to the sample surface, over a range of 2 θ from 10° to 60°, with a step width of 0.02° and data collection time of 5 s per step.

The elemental chemical surface composition of the PPAAm thin films were determined by high-resolution scanning X-ray photoelectron spectroscopy (XPS). The Axis Ultra DLD electron spectrometer (Kratos Analytical, Manchester, UK) runs with monochromatic Al Kα radiation (1486 eV; 150 W), implemented charge neutralization, and a pass energy of 80 eV for estimating the chemical elemental composition. The spot size was 250 µm. On each sample, three spots were measured and averaged in different positions. The takeoff angle was 90° in all cases.

Data acquisition and processing was carried out with the “vision 2.1.3” software (operating software Kratos) [13]. The labeling of primary amino groups was performed with 4-trifluoromethyl-benzaldehyde (TFBA, Alfa Aesar, Haverhill, MA, USA) at 40 °C over a saturated gas phase of 2 h. The density of the amino groups NH_2_/C was determined from the fluorine elemental fraction [35].

The chemical composition and molecular structure of PPAAm thin films were analyzed by means of FT-IRRAS (FT-IR Type: Spectrum One, PerkinElmer, Waltham, MA, USA) [36]. The spectra were obtained at the spectral resolution of 4 cm^−1^, with the number of scans at 32 in the wavenumber region 4000 to 600 cm^−1^.

#### 2.3.2. Film Thickness, PPAAm Roughness

The thickness of the deposited films was determined by surface profilometry. The film thickness can be measured solely on very smooth surfaces; therefore, silicon wafers were used and coated in the same plasma process together with the ceramic samples. For this purpose, before coating, very thin strips of cellulose acetate were deposited across the substrate surfaces. After film deposition, the acetate strip was removed. The height of the step obtained between the coated and uncoated surface area was determined with the help of the surface profiler (Dektak3ST, Veeco Instruments, Santa Barbara, CA, USA). Every sample was measured at a minimum of three different positions. Tests by spectroscopic ellipsometry (SE 850 spectroscopic ellipsometer, SENTECH Instruments, Berlin, Germany) confirmed the correctness of this method.

Surface roughness was determined using the scanning probe microscope diCP2 (Veeco Instruments) in non-contact mode (cantilever MPP111, Veeco Instruments) with a tip radius of 10 nm before and after PPAAm film deposition on silicon wafers (Universitywafers.com). The roughness Rq was calculated on a 10 µm × 10 µm wide area.

#### 2.3.3. Surface Free Energy, Contact Angle, Zeta Potential

The surface energy with its polar and disperse components was calculated from measurements of contact angles of three different liquids: Water, ethylene glycol, and methylene iodide. The contact angles were measured by the sessile drop method (drop volume ~0.5 μL) using the measuring system OCA30 (Data Physics Instruments GmbH, Filderstadt, Germany) and the software SCA 20. The surface energy was calculated using the methods of Owens, Wendt, and Rabel [37,38]. These measurements were always performed within 30–60 min after sample preparation at five different positions along the ceramic surface [39].

Existing surface charges can be estimated by determining the so-called zeta potential. Streaming potential measurements were carried out for various pressures using the Electrokinetic Analyzer SurPass (Anton Paar Germany GmbH, Ostfildern, Germany) to determine the streaming potentials dependent on the pressure. The measurements were performed in a 0.001 mol/L KCl solution ranging from pH 6.0 to 8.0 with a gap height of 100 μm. The streaming current was determined depending on the pressure (max. 300 mbar). Finally, the surface charge was calculated according to the method of Helmholtz–Smoluchowski. Measurements were performed in quadruplicate on two independent samples.

### 2.4. Cell Culture Experiments

The experiments were performed with the human osteoblast-like cell line MG-63 (CRL1427™, ATCC^®^, American Type Culture Collection, Manassas, VA, USA). MG-63 cells are a suitable in vitro-model for basic bone-related research, as the cell morphology, expression of adhesion receptors (e.g., α1-5, αv, β1-3, β5, CD44, syndecans), cell cycle phases (G0, S, G2/M), and intracellular signaling molecules (e.g., p-p38 MAPK, pERK, p-Akt, and intracellular Ca^2+^) are stable over the passages 5 to 30, as Staehlke et al. discovered recently [40].

Titanium: For the experiments with Ti samples, the cells were cultured in Dulbecco’s Modified Eagle’s Medium (DMEM) (Life Technologies, Thermo Fischer Scientific, Waltham, MA, USA, #31966021) containing 10% fetal calf serum (FCS) (Biochrom FCS Superior, Merck KGaA, Darmstadt, Germany, #S0615) and 1% gentamicin (Ratiopharm GmbH, Ulm, Germany).

Ceramic: For the experiments with ceramic samples, the cells were cultured in DMEM (high glucose, Gibco Invitrogen, Karlsruhe, Germany), with 10% FCS (Gold, PAA, Pasching, Austria) for passaging or serum-free for the experiments and 1% gentamicin. Serum-free medium was used to avoid masking of the PPAAm-coated ceramic surface with proteins (soluble fibronectin) as described earlier [18]. The culture conditions were at 37 °C in a humidified atmosphere with 5% CO_2_.

#### 2.4.1. Cell Morphology

Titanium: The morphology of MG-63 cells on Ti samples after 1 h cell culture (30,000 cells/1 × 1 cm-wafer, in DMEM + 10% FCS) was analyzed by field emission scanning electron microscopy (FE-SEM, Merlin VP compact, Carl Zeiss Microscopy GmbH, Jena, Germany). The samples were fixed with 2.5% glutaraldehyde (Merck KGaA, Darmstadt, Germany) at 4 °C for at least 1 h. Samples were rinsed with 0.1% sodium phosphate buffer, dehydrated through a graded series of acetone (30% 5 min, 50% 5 min, 75% 15 min, 90% 10 min, 100% 2 × 10 min), and dried in a critical point dryer (K 850, EMITECH, Taunusstein, Germany), then gold sputtered with a coater (20 nm, SCD 004, BAL-TEC AG, Balzers, Liechtenstein). To image the cell morphology on the FE-SEM, an acceleration voltage of 5 kV, and a high-efficiency secondary electron detector were used.

Ceramic: The morphology of MG-63 cells on ceramic samples (series no. BK050, BK052) after 24 h of cell culture (serum-free DMEM) was examined using the SEM DSM 960A (Carl Zeiss). The samples were fixed with 4% glutaraldehyde (1 h), dehydrated through a graded series of acetone, dried in the critical point dryer, and sputtered with the coater as described above (see also patent, Chapter 5).

#### 2.4.2. Actin Cytoskeleton

Titanium samples: The actin cytoskeleton organization (30,000 cells/well) was observed using the inverted confocal laser scanning microscope LSM 780 (Carl Zeiss Microscopy GmbH, Jena, Germany) equipped with an He–Ne laser (excitation: 543 nm), and ZEISS 63× Plan Neofluar oil immersion objective (1.25 oil/0.17). The MG-63 cells were grown on Ti samples for 1 h (DMEM + 10% FCS), and then fixed with 4% paraformaldehyde (PFA, Sigma-Aldrich, St. Louis, MO, USA) at room temperature (RT) for 10 min and permeabilized with 0.1% Triton-X 100 (Merck) at RT for 10 min. For actin staining, cells were incubated with phalloidin-tetramethyl-rhodamine (TRITC, dilution 1:15 in phosphate buffer saline, Sigma-Aldrich) in the dark at RT for 30 min. Samples were embedded in the mounting medium Fluoroshield™ (Sigma-Aldrich) on a cover slip and stored in the dark at 4 °C. The software ZEN 2.3 software (ZEISS Efficient Navigation, ZEN 2.3, black edition, Carl Zeiss) was used for image acquisition.

Cell spreading: To quantify the cell area (in µm^2^), we measured 40 TRITC-stained cells/sample using the software ImageJ 1.48a (Wayne Rasband, National Institutes of Health, Bethesda, MD, USA).

#### 2.4.3. Cell Adhesion

Ceramic: Suspended MG-63 cells in serum-free DMEM were seeded onto the ceramic plates (Ø 20 mm; series no. BK034) at a density of 1 × 10^4^/specimen for 10 min, and non-adherent cells in the supernatant were counted and analyzed by flow cytometry (FACSCalibur, software CellQuest Pro 4.0.1, BD Biosciences, Franklin Lakes, NJ, USA). Cell adhesion was then calculated as a percentage of the cell number at 0 min. Three independent experiments were carried out.

### 2.5. Statistics

Titanium samples: Statistics were performed by GraphPad Prism 7 software for Windows (GraphPad Software Inc., La Jolla, CA, USA). The spreading data (not normally distributed) were evaluated by non-parametric Kruskal–Wallis test post uncorrected Dunn’s test.

Ceramic samples: The cell adhesion data were statistically evaluated by SPSS software 14.0 for Windows (SPSS Inc., Chicago, IL, USA). The differences were evaluated with Student’s *t*-test.

All: In general, the normal distribution was established using the Kolmogorov–Smirnov test. Data were presented as mean ± standard error of mean (s.e.m.). Statistical significance was established at *p* ≤ 0.05.

## 3. Results and Discussion

Plasma polymerized allylamine was deposited and investigated by us on a variety of materials (see Table 1). In recent years, we extended our work to anorganic oxide ceramic materials that should replace metallic implants for persons with allergies. So far, the influence of the plasma radiation on these ceramics was unknown.

The ceramic utilized for dental implants is a special yttrium-stabilized, tetragonal zirconia polycrystal (Y-TZP). The tetragonal crystal structure must be guaranteed for dental applications. It is known that the tetragonal crystallites can be transformed into their monoclinic modification under a volume expansion of ca. 3–5% followed by crack formation or break off of smaller particles; this can happen, for instance, via the influence of manual grinding or chemical manipulations [41,42]. Transformation toughening is responsible for the unique failure behavior of Y-TZP. Mechanical and/or chemical manipulations after sintering influence local phase transformations near the surface and reduce the toughness and crack resistance of the ceramic. The stability of the tetragonal crystal phase seems to be a problem [41].

Our aim was to determine both the applicability of the PPAAm layer on these crystalline zirconia ceramics and also the minimal possible film thickness that would allow cells to spread optimally. We carried out the standard plasma deposition process in dependence on the treatment time and described, among other things, the interaction of MG-63 cells with the PPAAm film deposited in different thicknesses on the Y-TZP ceramic.

### 3.1. Physico-Chemical Surface Properties of PPAAm-Treated Y-TZP Ceramic

As a precondition for our further investigations, we studied the crystallographic structure of the ceramic substrate by X-ray diffraction analysis before and after plasma treatment to determine the influence of the plasma process.

**Figure 4:**
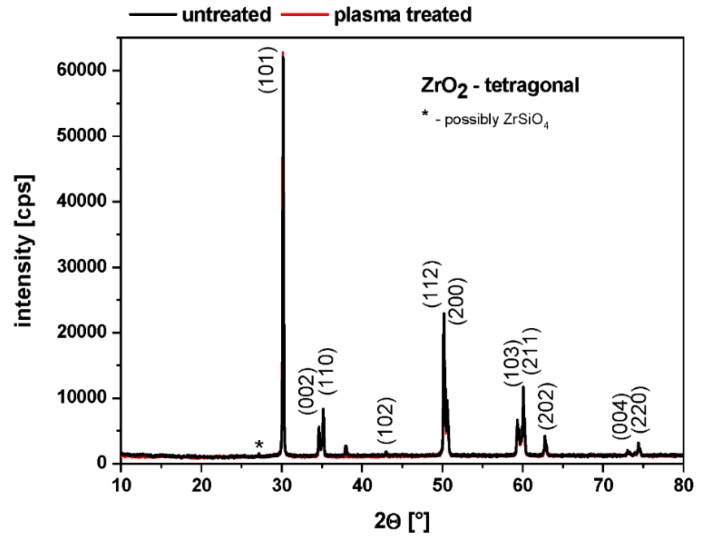
Grazing incidence X-ray diffraction (GIXRD) patterns of untreated and plasma polymerized allylamine (PPAAm) plasma-treated (960 s gross) tetragonal zirconia polycrystal (Y-TZP) ceramic.

The GIXRD analysis of the untreated and PPAAm-treated ceramic samples confirmed clearly, in agreement with the data file JCPDS 42-1164, the tetragonal crystal modification of Y-TZP (Figure 4). The undesired monoclinic phase was not detectable. The MW plasma process for the deposition of PPAAm has no influence on the tetragonal crystal structure of Y-TZP present.

The chemical composition and molecular structure of PPAAm thin films on ceramic (not shown) could be confirmed by means of FT-IRRAS. The FT-IRRAS spectra of PPAAm showed a high retention of the structural properties of the monomer allylamine H_2_C=CH-CH_2_-NH_2_ for the MW plasma deposition method used here in accordance with [13].

**Figure 5:**
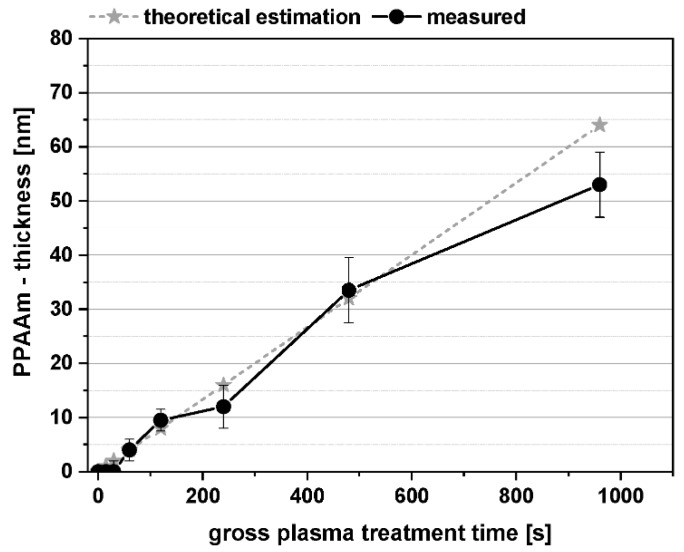
PPAAm film thickness dependent on the plasma treatment time determined on Si-wafers.

A further approach in our investigations was the gradual reduction of the PPAAm film thickness. We searched for the minimal possible film thickness, which would allow cells to behave optimally. For all samples (Si-wafer), one and the same standard plasma process parameter was used, only the gross treatment times varied (see Section 2.2) and, therefore, the film thickness. A quasi-linear film growth could be found from 15 s until about 480 s (Figure 5). At 960 s, etching processes more frequently accompany the deposition process, followed by a greater degree of cross-linking of the plasma polymer, reduced film thickness, a loss of surface functional groups, and increasing WCA. 

Layer formation and growth of the plasma polymer film PPAAm are of particular interest and can be explained by the results of Michelmore et al. [9] and Akhavan et al. [23]. They shed some light on the growth mechanisms of the very first layers of plasma polymer films, also for PPAAm. Michelmore et al. [9] verified a very smooth PPAAm film growth, and a continuous layer formation even for very thin films (<2 nm) from the very earliest stages on a silicon surface. The double bond in the precursor allylamine encourages a better cross-linking in PPAAm and seems to contribute to the smooth layer formation. Island-like structures could be excluded for PPAAm. These results confirm our observations of very smooth PPAAm films (480 s: Rq = 1.9 ± 0.2 nm; 960 s: Rq = 3.8 ± 0.9 nm). Akhavan et al. were, again, able to demonstrate that the formation of metallic (zirconium/titanium-) carbide and oxycarbide bonds in the initial stages of the film formation led to the excellent film adhesion of the plasma polymer. A 2D-like, layer-by-layer (Frank-van der Merve mechanism) manner of growth was observed [23].

Plasma-treated surfaces are very reactive upon completion of the plasma deposition process. So-called post plasma processes, especially oxidation and hydrolysis, take place after storage in ambient air, influencing the wettability and surface energy by changing the surface chemical compositions. The wettability is determined by the charge and polarity of the surface functional groups. Therefore, the water contact angle (WCA) measurements were always carried out 30–60 min after the plasma process in each case, to get comparable results.

**Figure 6:**
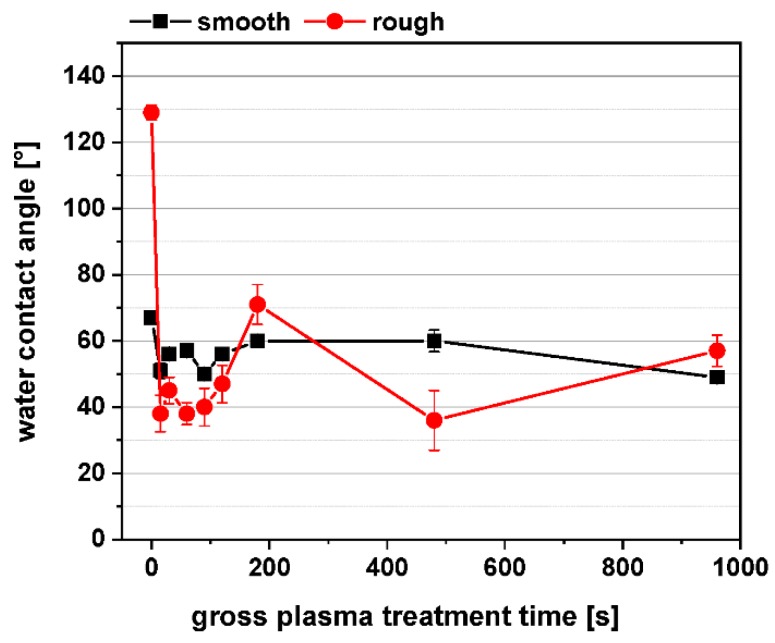
Water contact angles of untreated (time point 0 s) and PPAAm-coated smooth and rough ceramic. Note: Even a very short plasma treatment time of 15 s is sufficient to shift the water contact angles (WCA) of the untreated samples into the hydrophilic region. (Plasma treatment times: 15, 30, 60, 90, 120, 180, 480, and 960 s; *n* = 5).

Figure 6 shows the WCA of untreated (time point 0 s) and PPAAm-coated smooth and rough ceramic surfaces as a function of the plasma treatment time. In the case of the smooth ceramic (Rq ~273 ± 40 nm), the WCA are in a range between 50° and 60° (54.9 ± 4.4°), whereas the WCA values of the rough ceramic (Rq~1320 ± 20 nm) vary more strongly between 40° and 60° (46.5 ± 12°) and are more hydrophilic than the smooth ceramic surfaces. For both roughnesses, the WCA are in the hydrophilic region in which the wettability is optimal for cell responses [24]. Even a very short plasma treatment time of 15 s is sufficient to shift the WCA of the untreated samples into the hydrophilic region, here especially apparent for the rough surfaces.

The high WCA values of the untreated rough ceramic may be contributed to the enlargement of the effective surfaces area combined with higher hydrocarbon content. Our recent experiments by Rebl et al. [15] using polished and machined Ti showed similar tendency concerning WCA and roughness: For Ti-P (Ra 0.045 µm) and Ti-M (Ra 0.315 µm), the WCA values results in ca. 60° vs. 80°, respectively.

The zeta potential measured at pH 7 for the untreated rough zircon oxide ceramic is strongly negative at −53.1 ± 1.4 mV. The PPAAm nanofilm (480 s; on Si-Ti wafer) led to a positively charged surface (pH 6) at +32.8 ± 12 mV immediately after the deposition process and +35.5 ± 2 mV after 180 days storage in ambient air. The trend is consistent with our results in [13].

**Figure 7:**
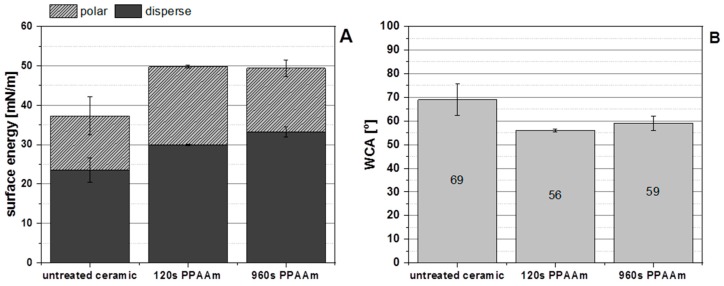
Surface energy (**A**) and water contact angle (**B**) of the untreated and PPAAm-coated smooth ceramic surface (*n* = 3). Note the tendency toward enhanced surface energy (dispersed and polar part) and a reduced water contact angle (i.e., higher hydrophilicity) on PPAAm.

The surface energy and water contact angle of untreated and PPAAm-coated (120 and 960 s) smooth ceramic surfaces demonstrated that the PPAAm treatment led to an enhancement of the total surface energy, including their polar and disperse parts (Figure 7A) and to decreasing WCA (Figure 7B). The total surface energy of the untreated smooth ceramic increased from about 37 ± 2 mN/m to about 50 ± 0.5 mN/m for both PPAAm coated surfaces (for 120 s and 960 s brutto, respectively). Also, the disperse and polar part of the surface energy increased on PPAAm. The disperse part of the untreated ceramic 24 ± 3 mN/m rose to 30 and 33 mN/m and the polar part of the untreated ceramic 14 ± 5 mN/m to 20 and 16 mN/m for 120 s and 960 s PPAAm. Furthermore, the shorter PPAAm treatment time of 120 s has a higher polar and lower disperse part than 960 s PPAAm. Possible reasons for this may be a lower influence of the etching processes or more surface functional groups through a lesser degree of cross-linking.

**Figure 8:**
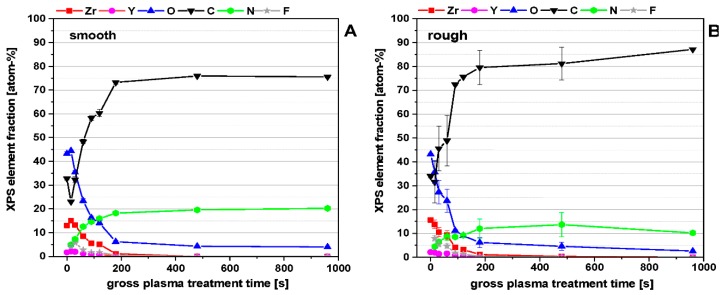
X-ray photoelectron spectroscopy (XPS) elemental content of PPAAm coating on a smooth (**A**) and rough (**B**) ceramic surface dependent on the treatment time. For a clearer presentation, the trace elements Al, Si, and Na were omitted.

The XPS surface composition, as a function of the plasma treatment time (Figure 8) for smooth and rough ceramic surfaces shows that, with increasing treatment time, the ceramic surface will be covered systematically by the plasma polymer film PPAAm. The elements of the ceramic substrate Zr, Y, in slight traces Al, Si, Na, and also O disappeared; meanwhile, the components of PPAAm C and N increased. A complete covering by PPAAm is reached at about 200 s. The significantly higher roughness of the rough ceramic led to higher standard deviations in C, O, and N. Zr and Y are in the same dimensions as is the case for smooth ceramic surfaces.

**Figure 9:**
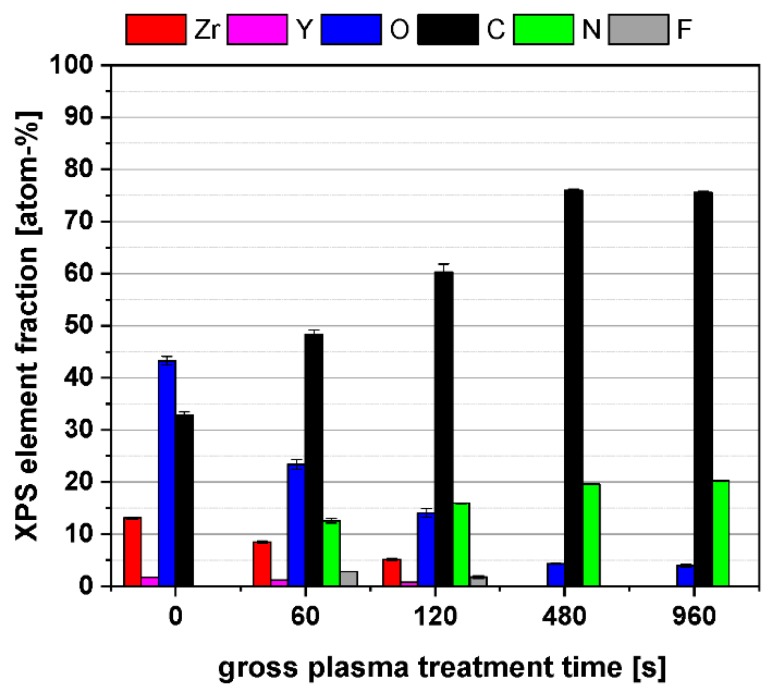
XPS elemental content after PPAAm coating of smooth ceramic dependent on the treatment time. The trace elements Al, Si, and Na were omitted.

The bar diagram (Figure 9) illustrates the surface composition of selected ceramic samples that were used in cell culture. The N/C ratios of PPAAm coatings for as little as 15 s is at 20% (not shown here) and increases continuously: at 60 s to 26%, 120 s to 26.4%, 480 s to 25.8%, and 960 s to 26.8%, whereby the theoretical N/C value of the precursor allylamine is 33%. The PPAAm films formed until about 120 s of treatment time already contain a considerable number of nitrogen functional groups at the surface.

Y-TZP ceramic surfaces that are negatively charged can be modified by the plasma polymer PPAAm under retention of the tetragonal crystal structure. Very smooth, positively charged PPAAm films could be deposited. In accordance with Michelmore et al. [9] and Akhavan et al. [23], a 2D-like, layer-by-layer (Frank-van der Merve mechanism) manner of growth can be assumed from the very earliest stages. Even short plasma treatment times led to PPAAm films with relatively high N/C ratios of about 20–26%. The WCA are optimal, lying in the hydrophilic range, and the total surface energy including their polar and disperse parts are enhanced on PPAAm. Thinner PPAAm films (<10 nm) seem to be beneficial in every respect and showed optimal conditions for adhesion and spreading of osteoblasts.

For future dental applications it is essential to evaluate the long-term stability of the PPAAm nanolayer on these Yttria-stabilized, tetragonal zirconia polycrystal ceramics (at least one year) as well as the in vivo effectiveness in the jawbone.

### 3.2. Cell Behavior on Titanium (Ti) and Ceramic Surfaces Bioactivated with Plasma Polymer (PPAAm)

**Figure 10:**
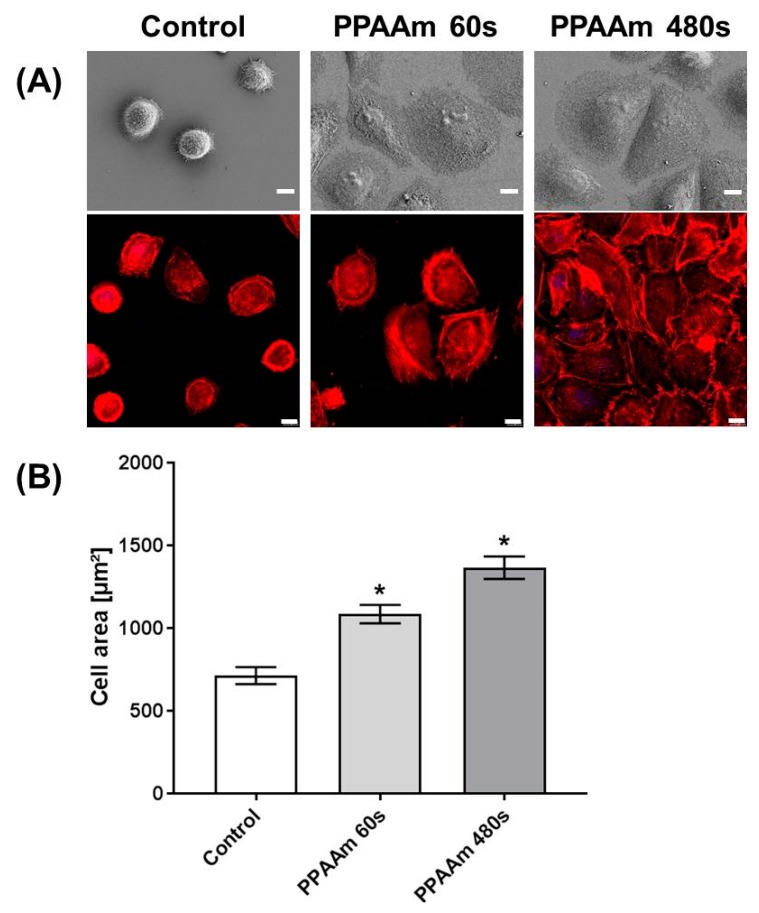
Impact of PPAAm plasma process duration and resulting nanolayer thickness on MG-63 cell morphology after 1 h on plane Ti. (**A**) Cell morphology (first row; FE-SEM Merlin VP compact, Carl Zeiss) and actin cytoskeleton (second row; LSM 780, Carl Zeiss); scale bars 10 µm. Note that the plasma process at 60 s gross is sufficient to significantly increase the cell area. (**B**) Cell area measurements (1 h) from the LSM actin images. (*n* = 40 cells, mean ± s.e.m., Kruskal–Wallis test post hoc uncorrected Dunn’s test, * *p* < 0.05 vs. control).

The plasma nanolayer PPAAm is convincing in its cell attractiveness. The 480 s plasma process on Ti, which results in a homogeneous nanolayer, allowed the cells to spread significantly in comparison with the uncoated Ti control, which we had already observed earlier [20]. In this work, it was of interest to determine whether a reduced plasma process time on Ti could induce the same spreading behavior. The PPAAm plasma treatment time of 60 s and 480 s gross, which corresponds to a layer thickness of about <10 nm and ~30 nm resp., revealed for both N/Cs ratios of about 26%. However, the cell-attractive characteristic of this nanolayer was obvious (Figure 10). After 1 h, cells on Ti PPAAm spread well and reached values of 1085.4 ± 55.5 µm^2^ and 1365.6 ± 68.7 µm^2^ for the PPAAm processes 60 s and 480 s, respectively (control: 713.8 ± 51.0 µm^2^). The actin cytoskeleton is already more pronounced in stress fibers after a 60 s deposition time of plasma polymerized allylamine.

**Figure 11:**
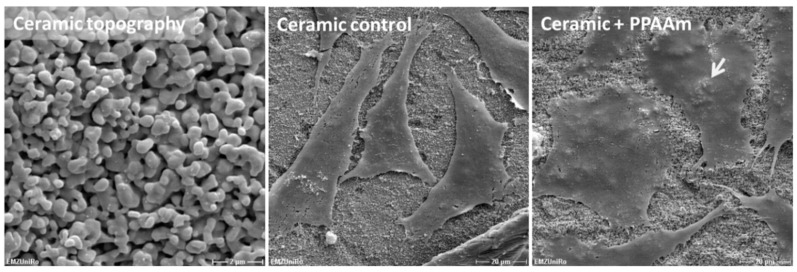
Cell morphology on plasma polymer (PPAAm)-modified rough ceramic after 24 h. The cell area on PPAAm (960 s gross) is increased and the cells melt into the underlying ceramic topography (arrow) in a way that the surface structures can be seen on the cell surface. (SEM DSM 960A, scale bars: left = 2 µm; middle and right = 20 µm, no. BK052).

**Figure 12:**
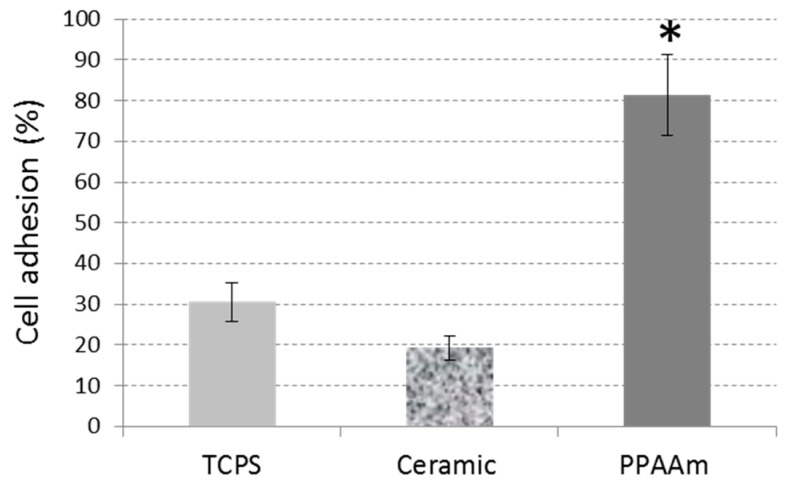
Initial cell adhesion (10 min) on plasma polymer modified smooth ceramic (PPAAm, 960 s gross). Flow cytometry, Student’s *t*-test: PPAAm vs. bare ceramic (no. BK034) * *p* < 0.01, PPAAm vs. tissue culture plastic (TCPS) * *p* < 0.05; mean ± s.e.m., *n* = 3 independent experiments.

Analogous to our investigations of PPAAm coatings on different materials (see Table 1), we prepared and analyzed the effects of conditioning ceramic surfaces with PPAAm, especially with regard to their further use as dental materials. The well-spread morphology also on PPAAm-coated ceramic surfaces is obvious (Figure 11). Ceramic on its own provides the cells with a surface characteristic, which leads to cell spread. In addition, the coating allows the cells to melt into the ceramic surface structure. The cells mimic the topography and the underlying structure appears through the flattened cell. These phenomena could also be observed with cells on metal surfaces, especially on corundum-blasted sharp-edged Ti coated with PPAAm, process duration 960 s gross resulting in a 50 nm layer [15]; or on plasma chemical oxidized surfaces additionally coated with PPAAm [11]. On PPAAm-modified ceramic, the cells are able to adhere immediately in the first 10 min and to a great degree of more than 80% compared with the untreated ceramic and the TCPS control at 81.4 ± 9.9%, 19.3 ± 2.9%, and 30.6 ± 4.7%, respectively (Figure 12).

Summarizing all results with ceramic surfaces as well as other biomaterials tested (see Table 1), we can say that the moderately positively charged plasma polymerized allylamine (PPAAm) nanolayer is very cell attractive. This phenomenon can be seen in many cell biological concerns: (i) On PPAAm-modified materials, the cell spreading is very fast, cells are flattened, and cells melt into the surface structure [15], and (ii) this cell attractiveness leads to the next phenomenon that cells “forget” the underlying surface structure. This could be observed for the first time by our group. Normally, on material topographies, cells feel the underlying topography, i.e., cells are aligned on micro-grooves or machined Ti. But if these materials additionally PPAAm-modified, the cells not only adhere better and spread faster, but also override the topography [10,15].

## 4. Conclusions

Yttria-stabilized tetragonal zirconia polycrystal (Y-TZP) ceramic surfaces can be modified by the plasma polymer PPAAm under retention of the tetragonal crystal structure. The ceramic surface is negatively charged. Very smooth, positively charged PPAAm films could be deposited and a two-dimensional-like, layer-by-layer manner of growth can be assumed from the very earliest stages. Even short plasma treatment times led to PPAAm films with relatively high N/C ratios of about 20–26%. The WCA are in the optimal hydrophilic range. The surface energy is enhanced at ~50 mN/m independent from the plasma deposition time (120, 960 s) on ceramic. XPS revealed that components of PPAAm C and N reached their maximum already after a short plasma process. Thinner PPAAm films (<10 nm) seem to be beneficial in every respect and showed optimal conditions for adhesion and spreading of human osteoblasts. Finally, we suggest that PPAAm as a cell-attractive nanolayer is also suitable for the bioactivation of dental implant surfaces.

## 5. Patent

A process for preparing ceramic implants for medical purposes. European patent specification. Vita Zahnfabrik H. Rauter GmbH & Co. KG, 79713 Bad Saeckingen (DE). Date of publication and mention of the grant of the patent: 22.03.2017, Bulletin 2017/12, Application number: 12753522.7, Date of filing: 05.09.2012. International application number: PCT/EP2012/067286. US14/236,740, US9, 241, 795, 26.01.2016.

## Figures and Tables

**Table 1:** Overview of plasma polymerized allylamine (PPAAm) surface coating of diverse bulk materials, their physico-chemical characteristics, and cell biological outcomes [10,11,12,13,14,15,16,17,18].

Materials	Chemistry	WCA (^o^)	Zeta Potential (Mv)	XPS (%)	Surface Energy (mN/m)	In Vitro Cell Behavior	Published
Silicon-titanium microgrooves, 20 × 2 µm (ZfM)	PPAAm; methyl-carboxyl- plasma polymer, COL-I, RGD; 480 s gross	57°	**+8.6** at pH 7.4	N/C 31 O/C 4	‒	Abrogated MG-63 cell contact guidance; randomly oriented actin stress fibers, PPAAm vs. all coatings	Moerke C et al., ACS Applied Material Interfaces, 2017 [10]
Ti-6Al-4V plasma chemical oxidation (PCO)	PPAAm; 60 s gross, 960 s gross	57–60°	positively charged, by AFM	N/C 32 O/C 2 NH_2_/C 2.8	polar 16 disperse 30 total 46	Accelerated MG-63 cell-surface interlocking, actin formation around the pores of porous ceramics; 3-fold increase of cell area (30 min)	Rebl H et al., Materials Science and Engineering C, 2016 [11]
β-tricalcium phosphate hybrid scaffolds	PPAAm; 960 s gross	‒	‒	N/C 29 O/C 10 NH_2_/C 2.5	‒	Continuous cellularization of hybrid 3D scaffolds (14 days); DNA concentration at the bottom 7.7-fold higher; MG-63 cell migration enhanced	Bergemann C et al., Materials Science and Engineering C, 2016 [12]
Ti-6Al-4V, cp, grade 2, polished	PPAAm; 960 s gross	47° after prep. 40–50° after 360 days storage on air	**+13.9** (2 days) **+20.9** (20 days) **+26.3** (200 days) at pH 6	N/C 27 O/C 6 NH_2_/C 2.5	polar 25 disperse 29 total 54	Aging and γ-sterilization of PPAAm; maintenance of cell adhesion capacity up to 360 days 2.2-fold and 1.6-fold increased cell areas (30 min, 24 h, resp.); cell area still visibly larger after 360 days of storage in air (5, 10, 30, 60 min, 24 h)	Finke B et al., Langmuir, 2014 [13]
Electrospun poly(l-lactide-co-d/l-lactide) mesh	PPAAm; (preactivation in Ar/O_2_ plasma) 480 s gross	5°	‒	N/C 28 O/C 5 NH_2_/C 2.5	polar 40 disperse 28 total 68	SV40-HUC-1 uroepithelial cells, Ca9-22 gingiva epithelial cells: enhanced cell integration in PLA fiber meshes; SV40-HUC-1 cell area: 1.26-fold, Ca9-22 cell area: 1.35-fold (both 30 min), spreading not influenced by γ-sterilization	Schnabelrauch M et al., International Journal of Polymer Science, 2014 [14]
Ti, cp, grade 2, P—polished, M—machined, CB—corund. blasted	PPAAm; PEG DA-COL-I, GDA-COL-I, 960 s gross	47° P-PPAAm 56° M-PPAAm 41° CB-PPAAm	‒	‒	P-PPAAm total 55 M-PPAAm total 50 CB-PPAAm total 57	Impact of plasma chemistry versus Ti surface topography: MG-63 cells literally melt into the groove structure; fewer elongated cells; 4.7-fold increased cell adhesion (5 min); 2.3-fold increased cell area (30 min)	Rebl H et al., Acta Biomaterialia, 2012 [15]
Borofloat glass	PPAAm; COL-I; 960 s gross	50°	**+14** at pH 6	N/C 20 O/C 11 NH_2_/C 2–3	‒	Enhanced vinculin mobility (1.5-fold, nm/min) in vital MG-63 cells; 1.5-fold increased vinculin contact length; 3.7-fold increase of cell area (30 min); all vs. COL-I	Rebl H et al., Advanced Engineering Materials, 2010 [16]
Bionas^®^ 2500 metabolic sensor chips SC 1000	PPAAm; 120 s gross	45°	**+14** at pH 6	N/C 20 O/C 14	polar 27 disperse 27 total 54	MG-63 cell coverage of chips 2-fold, 72%; cell area increased 1.9-fold (4 h); vital cell adhesion significantly higher (0–24 h), e.g., 2.31-fold (2 h); acidification and oxygen consumption not influenced	Rebl H et al., International Journal of Artificial Organs, 2010 [17]
Ti, cp, grade 2 polished	PPAAm, PEG DA-COL-I, COL-I; 960 s gross	48°	**+7.7** at pH 6	N/C 28 O/C 4 NH_2_/C 2.5	‒	Hyaluronan-mediated MG-63 cell adhesion; cell adhesion increased 7-fold (15 min); accelerated formation of actin cytoskeleton and paxillin and vinculin (60 min), vs. Ti-P, comparable with collagen coatings	Finke B et al., Biomaterials, 2007 [18]
